# Single-Dose Lignocaine-Based Blood Cardioplegia in Single Valve Replacement Patients

**DOI:** 10.21470/1678-9741-2016-0025

**Published:** 2017

**Authors:** Jaydip Ramani, Amber Malhotra, Vivek Wadhwa, Pranav Sharma, Pankaj Garg, Malkesh Tarsaria, Himani Pandya

**Affiliations:** 1 Department of Cardiovascular and Thoracic Surgery of U.N. Mehta Institute of Cardiology and Research Center (Affiliated to B.J. Medical College), Civil hospital Campus, Asarwa, Ahmedabad, India.; 2 Department of Cardiovascular and Thoracic Surgery of U.N. Mehta Institute of Cardiology and Research Center (Affiliated to B.J. Medical College), Civil hospital Campus, Asarwa, Ahmedabad, India.

**Keywords:** Cardiopulmonary Bypass, Cardioplegic Solutions, Lidocaine, Heart Valves/Surgery, Aortic Valve/Surgery, Mitral Valve/sSurgery

## Abstract

**Objective:**

Myocardial protection is the most important in cardiac surgery. We compared our modified single-dose long-acting lignocaine-based blood cardioplegia with short-acting St Thomas 1 blood cardioplegia in patients undergoing single valve replacement.

**Methods:**

A total of 110 patients who underwent single (aortic or mitral) valve replacement surgery were enrolled. Patients were divided in two groups based on the cardioplegia solution used. In group 1 (56 patients), long-acting lignocaine based-blood cardioplegia solution was administered as a single dose while in group 2 (54 patients), standard St Thomas IB (short-acting blood-based cardioplegia solution) was administered and repeated every 20 minutes. All the patients were compared for preoperative baseline parameters, intraoperative and all the postoperative parameters.

**Results:**

We did not find any statistically significant difference in preoperative baseline parameters. Cardiopulmonary bypass time were 73.8±16.5 and 76.4±16.9 minutes (*P*=0.43) and cross clamp time were 58.9±10.3 and 66.3±11.2 minutes (*P*=0.23) in group 1 and group 2, respectively. Mean of maximum inotrope score was 6.3±2.52 and 6.1±2.13 (*P*=0.65) in group 1 and group 2, respectively. We also did not find any statistically significant difference in creatine-phosphokinase-MB (CPK-MB), Troponin-I levels, lactate level and cardiac functions postoperatively.

**Conclusion:**

This study proves the safety and efficacy of long-acting lignocaine-based single-dose blood cardioplegia compared to the standard short-acting multi-dose blood cardioplegia in patients requiring the single valve replacement. Further studies need to be undertaken to establish this non-inferiority in situations of complex cardiac procedures especially in compromised patients.

**Table:** 

Abbreviations, acronyms & symbols	
AF	= Atrial fibrillation
CMIA	= Chemiluminescent microparticle immunoassay
CPB	= Cardiopulmonary bypass
CPK-MB	= Creatine-phosphokinase-MB
ICU	= Intensive care unit

## INTRODUCTION

Myocardial protection in its largest definition includes all drugs and techniques used to maintain or to protect the cellular integrity of the myocardial cell against the devastating effect of oxygen deprivation. The main principles of myocardial protection are reduction of metabolic activity by hypothermia and therapeutic arrest of the electrical activity and contractile apparatus of the myocytes by administering cardioplegic solution (*e.g.*, depolarization of the membrane potential by high potassium blood cardioplegia^[1-3]^.

Myocardial protection against global ischemia-reperfusion injury during open-heart surgery with cardiopulmonary bypass (CPB) and cardioplegic arrest remains a challenging problem. A recent survey of UK practice found that 56% of surgeons use cold blood cardioplegia, 14% use warm blood cardioplegia, 14% use crystalloid cardioplegia, 21% use retrograde infusion and 16% do not use any cardioplegia^[4]^.

Short-acting blood cardioplegia requires repetition at every 20 minutes with interruption of ongoing surgical procedure. Crystalloid cardioplegia is repeated at every 60 minutes but it causes significant hemodilution. Therefore, to come over these problems, we modified our blood cardioplegia which has advantage of both blood and crystalloid cardioplegia.

We modified our routine standard blood cardioplegia with additives over period of time and we have been using this modified cardioplegia as a single-dose long-acting blood cardioplegia over many years. In this study, efficacy of modified long-acting lignocaine-based blood cardioplegia solution on myocardial protection was compared with the short-acting St Thomas I blood-based cardioplegia in patients undergoing single valve (mitral or aortic) replacement surgery.

## METHODS

### Study Population and Design

We conducted a prospective randomized controlled study of 110 patients who underwent single (aortic or mitral) valve replacement surgery between June 2013 and August 2015 in Department of Cardiovascular and Thoracic Surgery at U.N. Mehta Institute of Cardiology and Research Center , Ahmedabad, India, after taking consent of all patients and ethical committee approval. Patients were divided in two groups based on the cardioplegia solution used. In group 1 (56 patients), long-acting lignocaine-based blood cardioplegia solution was administered as a single dose while in group 2 (54 patients), standard St Thomas I B (short-acting blood-based cardioplegia solution) was administered and repeated every 20 minutes. We excluded two patients from group 2 who required modified Bentall's procedure. In all patients, cardioplegia was delivered by antegrade method with topical surface cooling of heart. We included the patients undergoing single valve (mitral or aortic) replacement surgery with or without tricuspid valve repair. Patients with severe left ventricular dysfunction (ejection fraction < 35%), double valve replacement, valve repair (mitral/aortic), emergency surgery, New York Heart Association class IV, associated coronary artery disease, and redo surgery were excluded from the study. Apart from preoperative baseline parameters, patients were compared for intraoperative parameters like coronary sinus blood sample analysis at 20, 40, 60 minutes, aortic cross clamp time, CPB time, rhythm disturbances and need for pacing. Patients were also compared for postoperative parameters like serum creatine-phosphokinase-MB (CPK-MB) and lactate at 6, 12, 24 and 48 hours, cardiac output at 24, 48 and 72 hours, arrhythmias, inotropic score, blood investigations on 1^st^, 3^rd^ and 7^th^ day, 2D echocardiography on 5^th^ day, intensive care unit (ICU) stay, hospital stay and postoperative complications.

### Anesthetic and Surgical Protocol

Anesthesia was induced with midazolam 0.1 mg/kg, fentanyl 5 µg/kg and vecuronium 0.1 mg/kg, glycopyrollate 40 µg/kg stat and maintained with sevofluorane, vecuronium, oxygen, and air and injection fentanyl during single valve replacement. Following induction, a Swan-Ganz thermistor-tipped catheter was inserted via internal jugular vein and remained *in situ* for 72 hours. All baseline values and hemodynamic data were recorded. Inotropes were prepared as per-protocol and postoperative inotropic score was calculated.

All operations were performed through midline sternotomy using CPB with extracorporeal circulation using aorto-bicaval cannulation and moderate hypothermia. Retrograde coronary sinus cannulation was done in all patients. Antegrade aortic root (or coronary ostial cardioplegia during aortic valve replacement) was delivered. Coronary sinus blood samples were collected from one port of retrograde cannula. In case of single-dose blood cardioplegia group, samples were also collected at every 20 minutes after flushing with small amount of cardioplegia solution.

### Myocardial Protection

#### Method of Preparation of Cardioplegia

- Single-dose long-acting blood cardioplegia: As mentioned in Table 1, constituents added in 200 ml Ringer lactate are 13 ml of sodium bicarbonate, 5 ml of potassium chloride, 16 ml of 20% mannitol, 4 ml of 50% magnesium sulphate, 7 ml of 2% lignocaine, 2 ml of dexamethasone, and then 800 ml blood added to it, thus making it a solution of 4:1 blood and crystalloid similar to St Thomas I B cardioplegia.
  **Table 1:** Cardioplegia composition.

Components	Group 1	Group 2
Base solution	200 ml Ringer lactate	200 ml normal saline
Sodium	140	142
Potassium	13 mEq	16 mEq
Sodium bicarbonate	24 mEq	24 mEq
Magnesium	24 mEq	16 mEq
Calcium	1 mmol/l	1.7 mmol/l
Osmolality	320 mOsm/l	310 mOsm/l
Steroid	Dexamethasone 16 mg	-
Lignocaine/procaine	130 mg 2% lignocaine	13.64 mg 1% procaine
20% mannitol	16 ml mannitol	-
Blood	800 ml of cold blood	800 ml of cold blood
Final composition	4 part blood:1 part cardioplegia	4 part blood:1 part cardioplegia
- Short-acting St Thomas I B cardioplegia: As mentioned in Table 1, constituents are added in 200 ml normal saline and then 800 ml blood added to it, thus making it a solution of 4:1 blood and crystalloid.
  - Group 1 - Single-dose lignocaine-based blood cardioplegia solution: Cardioplegia was given in dose of 30 ml/kg over 5-8 minutes with pressure of 80-100 mmHg and temperature of 20º C as single-dose cardioplegia.
  - Group 2 - Short-acting St Thomas I B cardioplegia: Cardioplegia was given in dose of 30 ml/kg over 5-8 minutes with pressure of 80-100 mmHg and temperature of 20º C and repeated every 20 minutes.

### Arrhythmias

Patients were compared for rhythm changes intraoperatively and postoperatively (up to 7^th^ day). New-onset postoperative atrial fibrillation (AF) was defined (STS Adult Cardiac Surgery Database) as AF occurring during hospitalization after cardiac surgery in a patient with no history of AF or if AF lasts for more than 10 minutes that requires medical treatment. Other arrhythmias including ventricular tachycardia (lasting for more than 30 seconds), ventricular fibrillation, hemodynamically significant premature contractions (more than grade 3 - multiform, repetitive, R on T pattern according to Lown grading) and post-operative heart block were also noted.

### Cardiac Biomarkers

CPK-MB enzymatic basal level was measured which was 20-25 U/l. It is measured by IFCC method/immunoinhibition performed on architect system (Abbott Laboratory). The upper normal reference limit (99^th^ percentile) was less than 24 U/l. Blood CPK-MB of > 125 U/l (five times the basal value) was considered to be significant. Serum cardiac troponin-I concentration was measured by chemiluminescent microparticle immunoassay (CMIA) on architect system (Abbott Laboratory). The upper normal reference limit (99^th^ percentile) was less than 0.03 ng/mL. Blood lactate of > 4 ng/dl was taken as significant value.

### Perioperative Myocardial Infarction

Two of the following 3 criteria had to be fulfilled to diagnose a perioperative myocardial infarction: 1) CK-MB and/or troponin-T elevation above normal limits. 2) Appearance of a new postoperative Q wave on the electrocardiogram of more than 0.03 second. 3) A new hypokinetic or akinetic area in the left or right ventricle by 2D echocardiography.

### Statistical Analysis

Statistical data were analyzed using SPSS version 22 software system. Continuous data were expressed as mean±SD. Univariate analysis of continuous data was performed using Chi-square test or through a Student's t-test. Level of significance was accepted at *P*<0.05.

## RESULTS

All preoperative baseline parameters were comparable (Table 1). There was no significant difference in the frequency of mechanical ventilation time 6.83±2.33 and 6.34±2.10 (*P*=0.63) minutes, hospital stay 8.63±3.20 and 8.08±2.52 (*P*=0.72) days, myocardial infarction, inotropic support score 6.3±2.52 and 6.1±2.13 (*P*=0.65), and/or 30-day mortality between the two groups. In this study, mean aortic cross clamp time in single-dose blood cardioplegia Group 1 was 58.9±10.3 min *vs.* 66.3±11.2 min (*P*=0.23) for ST 1 B Group 2. Mean CPB time in single-dose blood cardioplegia group was 73.8±16.5 min *vs.* 76.4±16.9 min (*P*=0.43) in ST 1 B Group 2. The cross clamp time was slightly shorter in the single-dose blood cardioplegia group owing to the repetitions required in the ST 1 B Group 2 as shown in Table 2. We did not find any statistical significant difference in aortic cross clamp and CPB time and its effect on intraoperative and postoperative recovery of cardiac rhythm and function. In coronary sinus blood samples pH, pCO_2_, pO_2_, sodium, potassium, glucose, hematocrit, calcium, lactate levels in both groups were comparable. No statistically significant differences were found between the two groups. We also did not find any statistical significant difference in postoperative serum CPK-MB, troponin I and lactate at 6, 12, 24 and 48 hours, cardiac output study at 24, 48 and 72 hours. All parameters were comparable (Tables 3 and 4).

**Table 2:** Patient characteristics.

Characteristics	Group 1	Group 2	*P* value
Age (years)	37±12	32±14	0.41
Females (%)	28 (50%)	36 (65%)	0.62
EuroSCORE 2	2.51±1.34	2.29±1.36	0.42
Preoperative serum creatinine (mg/dl)	1.02±0.3	1.12±0.4	0.19
Creatinine 48 hour postoperative (mg/dl)	0.93±0.28	0.90±0.12	0.48
Preoperative ejection fraction (%)	56.0±5.2	55.6±4.9	0.63
Aortic cross clamp time (min)	58.9±10.3	66.3±11.2	0.23
Cardiopulmonary bypass time (min)	73.8±16.5	76.4±16.9	0.43
Mechanical ventilation time (hrs.)	6.83±2.33	6.34±2.10	0.63
ICU stay (days)	6.39±2.17	6.76±2.64	0.74
Hospital stay (days)	8.63±3.20	8.08±2.52	0.72
Maximum inotropic score	6.3±2.52	6.±2.13	0.65
Postoperative new onset AF	5 (8.9%)	7 (12.9%)	0.12
Myocardial infarction	-	-	

AF = atrial fibrillation; ICU = intensive care unit

**Table 3:** Cardiac biomarkers.

CPK-MB (U/l)	Group 1	Group 2	*P* value
6 hours	62.9±27.6	69.1± 22.7	0.19
12 hours	59.6±27.4	61.01±22.6	0.24
1 day	52.7±20.3	55.3±21.3	0.21
2 day	41.6±19.7	46.4±19.1	0.75
**Serum troponin I (ng/ml)**			
6 hours	10.62±2.89	11.08±2.91	0.89
12 hours	7.08±2.34	7.32±2.10	0.78
1 day	4.63±1.09	4.92±1.20	1.23
2 day	2.46±0.42	2.86±0.56	1.20
**Serum lactate (ng/dl)**			
6 hours	6.0±1.4	6.3±1.2	0.06
12 hours	4.08±1.1	4.4±1.2	0.46
1 day	2.3±1.3	2.6± 1.5	0.77
2 day	1.7±0.3	1.9±0.5	0.62

**Table 4:** Cardiac output parameters.

Cardiac output parameters	Group 1	Group 2	*P* value
Cardiac index (l/min/m^2^)			
12 hours	3.33±0.6	3.38±0.5	0.66
24 hours	3.32±0.7	3.27±0.6	0.67
36 hours	3.44±0.5	3.45±0.5	0.87
**Left ventricle stroke work index (ml/m^2^)**			
12 hours	56.5±8.5	54.5±8.6	0.23
24 hours	53.1±9.1	54.4±9.8	0.15
36 hours	56.1±8.7	55.1±10.0	0.98
**Right ventricle stroke work index (ml/m^2^)**			
12 hours	4.2±1.14	4.2±1.2	0.95
24 hours	4.3±1.1	4.2±1.0	0.47
36 hours	4.34±0.9	4.2±0.8	0.52

## DISCUSSION

Lignocaine is classified as a sodium channel blocker and is class 1B antiarrhythmic. Intraoperative and postoperative arrhythmias were relatively less frequent but not statistical significant in single-dose cardioplegia group as compared with ST 1 B group. Blood cardioplegia is associated with significant reduction in postoperative arrhythmias as compared to crystalloid cardioplegia. Both our cardioplegias were blood-based. Leicher et al.^[5]^ in their study concluded that K^+^ induced increase coronary vascular resistance can be revented by lignocaine^[6-8]^.

There was no statistically significant difference for cross clamp and CPB times between the two groups. The shorter cross clamp times did not translate into any benefit in mortality or morbidity in terms of ventilation time, ICU stay or inotropic score.

Serum CPK-MB peaked in first 6 hours and started decreasing after 12 hours. CPK-MB levels of more than five times the basal value was taken as marker for perioperative myocardial infarction. It reached baseline level on 3^rd^ to 4^th^ day postoperatively. CPK-MB level was slightly higher in ST 1 B group than single-dose blood cardioplegia group. However, there was no statistically significant difference between the two groups. Long-term follow-up studies by Costa et al.^[9]^ and Klatte et al.^[10]^ have shown that patients with peak CK-MB release of more than 5 times the upper limit of normal continue to have a relatively high risk of mortality even after the first 30 postoperative days. However, in this study it never increased to 5 times above basal values^[11]^.

The serum troponin I release in the post-operative period was maximum at 6 hours with gradual decline at 48 hours. The enzyme release was less in single-dose blood cardioplegia group as compared to the ST 1 B group, but it was not found to be statistically significant. In this study, shorter cross clamp time and less release of myocardial injury markers found in single-dose blood cardioplegia group, but this did not translate into any clinical benefit, as there was no difference in both groups in terms of inotropic score, ventilation time or ICU stay. This can be attributed to exclusion of high-risk patients^[11]^.

Single-dose blood cardioplegia group had slightly lower levels of serum lactate as compared to ST 1 B group at all point of time. However, there was no statistically significant difference in serum lactate level in both groups. In this study, high levels were found 6 hours after surgery, which were normalized after 12 hours. Immediate higher levels were not related with any form of postoperative morbidity and mortality in our patients. There was no statistically significant difference between the two groups, providing evidence of equal efficacy of single-dose blood cardioplegia and multiple doses of ST 1 B. Lactate levels in this study corresponds to the accepted in value most of the other studies^[12,13]^.

Heart receives insult not only from inflammation caused by CPB, but also by repeated ischemia and reperfusions. The most potent anti-inflammatory substances are steroids. However, many studies demonstrated the role of steroids in reducing CPB related injury. Studies by Sellevold et al.^[6]^ suggested that inclusion of steroid improved the recovery of physiological indices, reduced CK leakage, increased myocardial ATP, reduced myocardial edema and prevented ischemia induced myocardial damage^[14]^. In the present study, we did not find any beneficial effect of steroid inclusion in our cardioplegia.

Myocardial edema also impairs myocardial function. Hyperosmotic mannitol scavenges free radicals and reduces myocardial cell swelling^[15]^. Magovern et al.^[16]^ demonstrated in rabbit heart preparations the superiority of mannitol (as compared to the glucose containing hyperosmolar solution and isosmolar solution) in improving in postischaemic ventricular function and reducing myocardial edema formation. There was no any statistical significant difference in ventricular function, inotropic score, and arrhythmias between the two groups. Inclusion of mannitol in this cardioplegia did not translate any statistical significant difference in selected patients with good ventricular functions. However, these constituents may provide their beneficial effects in patients with decompensated hearts.

We did not find any statistical significant difference in coronary sinus blood sample parameters.

Inclusion of additives like lignocaine, mannitol, and steroid did not offer any obvious benefit in prevention of various postoperative arrhythmias in study groups. They would have given extra benefits in patients with poor ejection fraction and high-risk patients. There was no any statistically significant difference between the two groups. Therefore, it was presumed that single-dose blood cardioplegia being a blood-based cardioplegia would be superior to other long-acting crystalloid cardioplegias in terms of postoperative arrhythmias. However, this can only be proved in a randomized control study comparing single-dose blood cardioplegia with other long-acting crystalloid cardioplegia.

Markers of myocardial function like cardiac index, left and right ventricle stroke work index at 12, 24 and 36 hours and 2D echocardiography was performed on 5^th^ postoperative day were comparable between both groups. The postoperative indices of left and right ventricle functions in this study were comparable to the other studies done for patients with preserved myocardial function^[17]^.

### Limitations

This study was a prospective randomized controlled study between single-dose lignocaine based-blood cardioplegia and multidose short-acting blood-based cardioplegia. Single-dose blood cardioplegia has proved to be equally safe and effective as short-acting ST 1 B cardioplegia in low-risk patients, single valve pathology and surgery requiring shorter cross clamp time.

1. This study included a relatively small sample size.
2. This study did not compare single-dose blood cardioplegia with other long-acting crystalloid cardioplegias. Further studies are required to compare the single-dose blood cardioplegia with other long-acting crystalloid cardioplegia in patients with complex cardiac procedures, which might require longer aortic cross clamp time.
3. Given the non-inferiority of single-dose blood cardioplegia now, further studies need to be undertaken to compare single-dose blood cardioplegia with St Thomas I blood-based cardioplegia in high-risk and compromised patients. Until then, St Thomas I blood-based cardioplegia will continue to be gold standard cardioplegia.

## CONCLUSION

This study proves the safety and efficacy of long-acting lignocaine-based single-dose blood cardioplegia compared to the standard short acting multidose blood cardioplegia in patients requiring the single valve replacement. Further studies need to be undertaken to establish this non-inferiority in situations of complex cardiac procedures especially in compromised patients.

**Table:** 

Authors' roles & responsibilities	
JR	Conception and design study; realization of operations; analysis and/or data interpretation; statistical analysis; manuscript redaction or critical review of its content; final manuscript approval
AM	Conception and design study; realization of operations; analysis and/or data interpretation; statistical analysis; manuscript redaction or critical review of its content; final manuscript approval
VW	Conception and design study; realization of operations; analysis and/or data interpretation; statistical analysis; manuscript redaction or critical review of its content; final manuscript approval
PS	Conception and design study; realization of operations; analysis and/or data interpretation; statistical analysis; manuscript redaction or critical review of its content; final manuscript approval
PG	Conception and design study; realization of operations; analysis and/or data interpretation; statistical analysis; manuscript redaction or critical review of its content; final manuscript approval
MT	Conception and design study; realization of operations; analysis and/or data interpretation; statistical analysis; manuscript redaction or critical review of its content; final manuscript approval
HP	Conception and design study; realization of operations; analysis and/or data interpretation; statistical analysis; manuscript redaction or critical review of its content; final manuscript approval

## References

[1] O'Brien JD, Howlett SE, Burton HJ, O'Blenes SB, Litz DS, Friesen CL (2009). Pediatric cardioplegia strategy results in enhanced calcium
metabolism and lower serum troponin T. Ann Thorac Surg.

[2] Oliveira MAB, Godoy MF, Braile DM, Lima-Oliveira APM (2005). Polarizing cardioplegic solution: state of the
art. Braz J Cardiovasc Surg.

[3] Sobrosa CG, Jansson E, Kaijser L, Bomfim V (2005). Myocardial metabolism after hypothermic retrograde continuous
blood cardioplegia with anterograde warm cardioplegic
induction. Braz J Cardiovasc Surg.

[4] Jacob S, Kallikourdis A, Sellke F, Dunning J (2008). Is blood cardioplegia superior to crystalloid
cardioplegia. Interact Cardiovasc Thorac Surg.

[5] Leicher FG, Magrassi P, LaRaia PJ, Derkac WM, Buckley MJ, Austen WG (1983). Blood cardioplegia delivery: deleterious effects of potassium
versus lidocaine. Ann Surg.

[6] Sellevold OF, Jynge P (1985). An experimental evaluation of glucocorticoid supplementation to
cardioplegic solutions in clinical use. Thorac Cardiovasc Surg.

[7] Dias RR, Dalva M, Santos B, Kwasnicka KL, Sarraff AP, Dias AR (2002). Influence of lidocaine on myocardial protection with blood
cardioplegia. Braz J Cardiovasc Surg.

[8] Dussin LH, Moura L, Gib MC, Saadi EK, Barbosa GV, Wender OCB (2008). Ultrastructural study of the myocardium using cardioplegic
crystalloid solution with and without procaine in patients undergoing aortic
valve replacement. Braz J Cardiovasc Surg.

[9] Costa MA, Carere RG, Lichtenstein SV, Foley DP, Valk V, Lindenboom W (2001). Incidence, predictors, and significance of abnormal cardiac
enzyme rise in patients treated with bypass surgery in the Arterial
Revascularization Therapies Study (ARTS). Circulation.

[10] Klatte K, Chaitman BR, Theroux P, Gavard JA, Stocke K, Boyce S (2001). Increased mortality after coronary artery bypass graft surgery is
associated with increased levels of postoperative creatine kinase-myocardial
band isoenzyme release: results from the GUARDIAN trial. J Am Coll Cardiol.

[11] Costa PS, Pereira SN, Moraes LB, Marques RS, Alvarez MAP, Daudt CAS (1988). Oxygenated cardioplegia in myocardial protection during cardiac
surgery: a clinical and enzymatic study. Braz J Cardiovasc Surg.

[12] Dobson GP, Faggian G, Onorati F, Vinten-Johansen J (2013). Hyperkalemic cardioplegia for adult and pediatric surgery: end of
an era?. Front Physiol.

[13] O'Blenes SB, Friesen CH, Ali A, Howlett S (2011). Protecting the aged heart during cardiac surgery: the potential
benefits of del Nido cardioplegia. J Thorac Cardiovasc Surg.

[14] Joudi M, Fathi M, Soltani G, Izanloo A (2014). Factors affecting on serum lactate after cardiac
surgery. Anesth Pain Med.

[15] Shinde SB, Golam KK, Kumar P, Patil ND (2005). Blood lactate levels during cardiopulmonary bypass for valvular
heart surgery. Ann Card Anaesth.

[16] Magovern GJ Jr, Bolling SF, Casale AS, Bulkley BH, Gardner TJ (1984). The mechanism of mannitol in reducing ischemic injury:
hyperosmolarity or hydroxyl scavenger?. Circulation.

[17] Mullen JC, Fremes SE, Weisel RD, Christakis GT, Ivanov J, Madonik MM (1987). Right ventricular function: a comparison between blood and
crystalloid cardioplegia. Ann Thorac Surg.

